# Supplementary material for: Saccharomyces boulardii (CNCM I-745) improves intestinal damage in sepsis by remodeling intestinal flora structure
Source: Front Cell Infect Microbiol. 2025 Jul 31;15:1602792. doi: 10.3389/fcimb.2025.1602792 (PMC12350329; doi:10.3389/fcimb.2025.1602792)
Supplement: Supplementary file 1 [file DataSheet1.zip › summary/summary/7_Differential_analysis/2_LEfSe_result/Group_DW0_vs_CW0_vs_BW0_vs_AW0_0.05_3.0/LEfSe_Group_DW0_vs_CW0_vs_BW0_vs_AW0.cladogram.pdf]

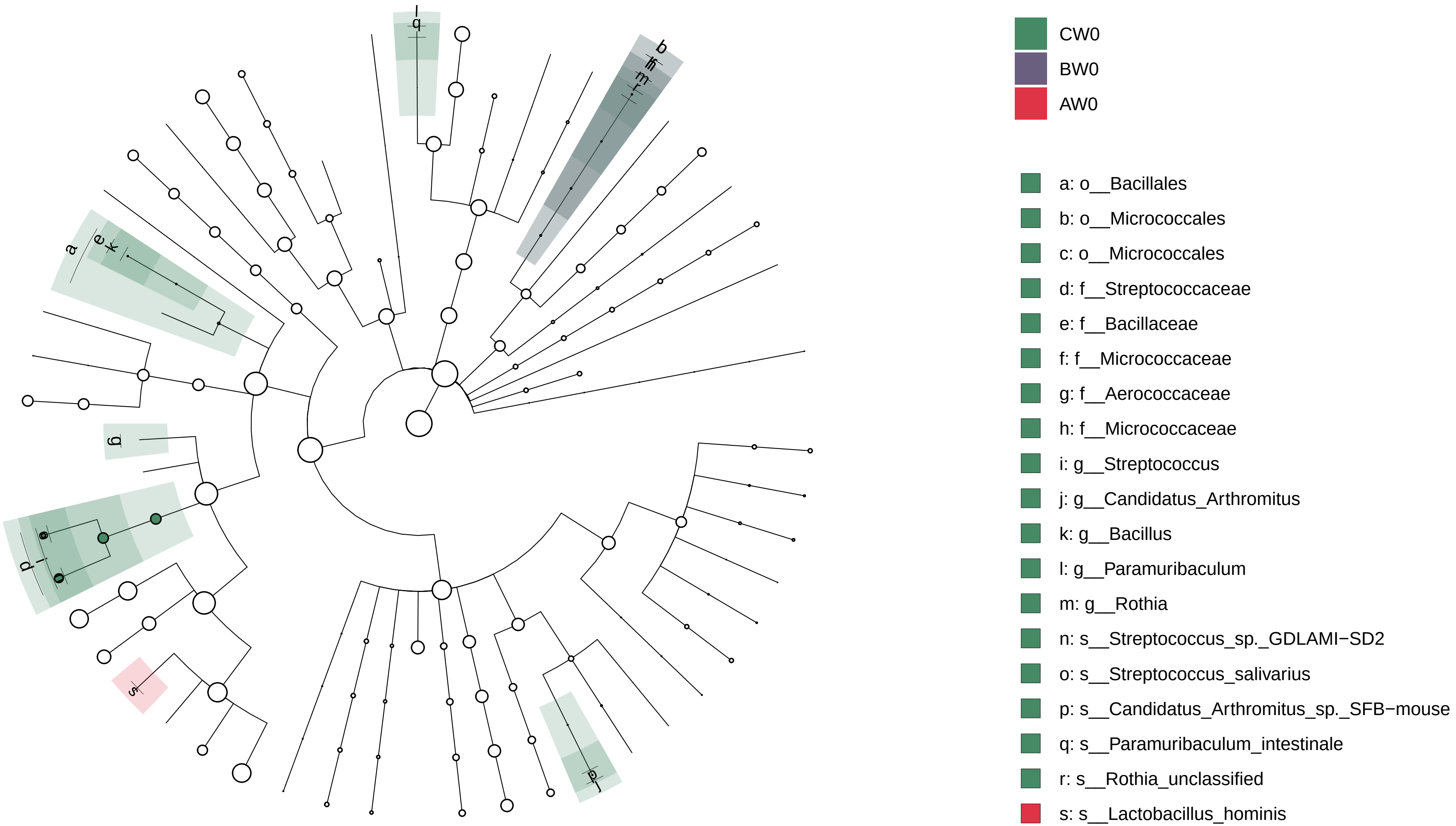
CW0
BW0
AW0
a: o__Bacillales
b: o__Micrococcales
c: o__Micrococcales
d: f__Streptococcaceae
e: f__Bacillaceae
f: f__Micrococcaceae
g: f__Aerococcaceae
h: f__Micrococcaceae
i: g__Streptococcus
j: g__Candidatus_Arthromitus
k: g__Bacillus
l: g__Paramuribaculum
m: g__Rothia
n: s__Streptococcus_sp._GDLAMI−SD2
o: s__Streptococcus_salivarius
p: s__Candidatus_Arthromitus_sp._SFB−mouse
q: s__Paramuribaculum_intestinale
r: s__Rothia_unclassified
s: s__Lactobacillus_hominis
